# Assessment of Chitosan/Gelatin Blend Enriched with Natural Antioxidants for Antioxidant Packaging of Fish Oil

**DOI:** 10.3390/antiox13060707

**Published:** 2024-06-11

**Authors:** Mia Kurek, Mario Ščetar, Marko Nuskol, Tibor Janči, Marija Tanksoić, Damir Klepac, Mojca Čakić Semenčić, Kata Galić

**Affiliations:** 1 Faculty of Food Technology and Biotechnology, University of Zagreb, HR-10000 Zagreb, Croatia; mscetar@pbf.hr (M.Š.); mnuskol@pbf.hr (M.N.); tjanci@pbf.hr (T.J.); marijat1998@gmail.com (M.T.); mojca.cakic@pbf.unizg.hr (M.Č.S.); kgalic@pbf.hr (K.G.); 2 Centre for Micro- and Nanosciences and Technologies, Faculty of Medicine, University of Rijeka, HR-51000 Rijeka, Croatia; damir.klepac@medri.uniri.hr

**Keywords:** gelatine, chitosan, natural antioxidants, gallic acid, essential oils, fish oil, active antioxidant packaging

## Abstract

In this research, bio-based films were developed using polyelectrolyte complexes derived from chitosan and gelatin for packaging fish oil. To further enhance the antioxidant functionality, the films were enriched with gallic acid and orange essential oils, either individually or in combination. Initially, the films were characterized for their physico-chemical, optical, surface, and barrier properties. Subsequently, the phenolic compounds and antioxidant capacity of the films were assessed. Finally, the films were tested as antioxidant cover lids for packaging fish oil, which was then stored at ambient temperature for 30 days, with periodical monitoring of oil oxidation parameters. This study revealed that the inclusion of gallic acid-induced possible crosslinking effects, as evidenced by changes in moisture content, solubility, and liquid absorption. Additionally, shifts in the FTIR spectral bands suggested the binding of gallic acid and/or phenols in orange essential oils to CSGEL polymer chains, with noticeable alterations in film coloration. Notably, films containing gallic acid exhibited enhanced UV barrier properties crucial for preserving UV-degradable food compounds. Moreover, formulations with gallic acid demonstrated decreased water vapor permeability, while samples containing orange essential oils had lower CO_2_ permeability levels. Importantly, formulations containing both gallic acid and essential oils showed a synergistic effect and a significant antioxidant capacity, with remarkable DPPH inhibition rates of up to 88%. During the 30-day storage period, fish oil experienced progressive oxidation, as indicated by an increase in the K232 value in control samples. However, films incorporating gallic acid or orange essential oils as active antioxidants, even used as indirect food contact, effectively delayed the oxidation, highlighting their protective benefits. This study underscores the potential of sustainable bio-based films as natural antioxidant packaging for edible fish oil or fresh fish, offering a promising tool for enhancing food preservation while reducing its waste.

## 1. Introduction

The development of edible films and coatings has made significant progress in recent decades and is expected to play a crucial role in future food packaging technologies. These films and coatings provide protection against physical, chemical, and biological contamination, extending the shelf life of food and reducing food waste [1]. As renewable and biodegradable alternatives, edible films and coatings offer a solution to the environmental problems caused by synthetic packaging materials [2]. To this end, a variety of bio-based polymers are being explored, primarily classified as polysaccharides and proteins from animal, plant, or microbial sources, including chitosan, gelatin, starch, cellulose, and pectins, among others [3]. The effectiveness of edible films depends on several factors, such as availability, structural barrier properties, optical properties, and sensory acceptability. Formulating a product that meets all requirements is a challenge due to the diverse nature and complex structures of the available biopolymers. Recent research has focused on edible two- and multi-component materials that offer improved functional properties. Composite films or coatings made by blending two or more film-forming substances exhibit superior physical, mechanical, and barrier properties compared to single-component materials. In addition, various active ingredients can be incorporated into these formulations to achieve antimicrobial and antioxidant effects or to improve sensory properties such as color and flavor [2,4,5,6,7,8,9]. The investigation of interpolymer interactions and the formation of polyelectrolyte complexes has become an important research focus due to its importance for both fundamental research and practical applications. Polyelectrolyte complexes are particularly attractive because they combine unique physico-chemical properties with high biocompatibility. These complexes are formed from the interaction between oppositely charged polyions, reducing the need for chemical cross-linking agents and thus minimizing the potential toxicity and undesirable side effects.

Several important properties of proteins and polysaccharides make them ideal for use as coatings and films in the food and pharmaceutical industries. In recent decades, chitosan (CS) has been of particular interest as a food-contact material and a carrier of natural substances due to its appealing properties, including an excellent gas and water vapor barrier, biodegradability, renewable properties, environmental friendliness, and low production costs. Chitosan films, however, have disadvantages, such as low mechanical stability, brittleness, and elasticity, and a high sensitivity to moisture, which limit their use [10,11].

Chitosan spontaneously forms polyelectrolyte complexes in solution when it combines with negatively charged polyions (below its pKa value) at low pH values (i.e., in dissolution media). In many cases, chitosan is bonded to gelatin (GEL), a water-soluble protein with remarkable characteristics, which forms homogeneous polyelectrolytes with chitosan through hydrogen bonding. Chemically active amino groups (–NH_2_) in flexible chains of CS react chemically with negatively charged carboxyl groups (–COOH) in GEL under acidic conditions to form polyelectrolytes [12,13]. A gelatin–chitosan complex exhibits excellent mechanical strength, barrier properties, and thermal stability, including antibacterial and antioxidant properties, and is effective at preserving practical foods (like meat, vegetables, and fruits) [14].

Although many previous studies have examined the physico-chemical properties of fish gelatin and its applications for composite films [15,16,17], films produced by stand-alone gelatin films still lack significant water resistance, particularly under high moisture conditions. There are many factors that can influence the process of producing a gelatin–chitosan complex, including the origin, molecular weight, deacetylation degree, pH, salts, concentration, temperature, active materials, modifiers, etc. [18], so it is still crucial to find modification strategies to overcome these disadvantages. The incorporation of bioactive compounds into biodegradable films is an effective active/intelligent packaging concept for extending shelf-life and maintaining or monitoring food quality and safety [19]. In addition, a crosslinking method with an appropriate crosslinker is crucial to overcome a protein-based polymer’s disadvantages. 

Previous research has demonstrated the positive effects of gallic acid (GA), a natural antioxidant, that acts as a crosslinker of various polymers including gelatin, chitosan, cellulose, and other hydrocolloids [20,21]. As a result of crosslinking with gallic acid, films containing chitosan and tuna lipid fractions [21] exhibited improved permeation and mechanical characteristics, with significantly reduced flexibility of the materials, even though their elastic modulus remained stable at elevated temperatures. Studies have shown that agricultural waste extracts, like citrus peels, pomaces, or similar, greatly improve the antioxidant activity of bio-based films, preventing lipid oxidation in food systems [22]. In addition, the essential oils from oranges, a by-product of orange juice production, were shown to be rich in flavonoids, polymethoxylates, and phytochemicals, as well as beneficial against some pathogenic bacteria [23,24]. Orange essential oils have compounds with bioactive potentials, and the antioxidant activities of various citrus species that can be used in the pharmaceutical and food industries have been reported previously [25,26]. Studies have indicated that sweet orange essential oils can exert antioxidant and antimicrobial effects not only through their major constituents (d-limonene) but also due to some other compounds’ combined action which makes differences among various citrus species [27,28]. Therefore, orange essential oils were shown to exhibit a free radical scavenging activity, about 50% inhibition for 1000 µg/mL, while when applied to food packaging, the DPPH scavenging rate of enriched chitosan films showed an inhibition from 48% to 58% [27] or it was successfully used to prolong the shelf life of fresh shrimps [29].

Fish oil is known for its high nutritional value, especially because of its high content of omega-3 polyunsaturated fatty acids (PUFAs), particularly eicosapentaenoic acid (EPA) and docosahexaenoic acid (DHA). These essential fatty acids play a crucial role in maintaining cardiovascular health, supporting cognitive function, and reducing inflammation. Regular consumption of fish oil has been linked to numerous health benefits, including lowering the risk of heart disease, improving mental health, and aiding in the management of inflammatory conditions such as arthritis [30,31]. Despite its significant health benefits, the oxidative stability of fish oil is a major concern. Fish oil is highly susceptible to oxidation due to its high PUFA content. Oxidation leads to the formation of undesirable compounds such as peroxides and aldehydes, which can affect the nutritional quality of the oil and cause an unpleasant taste and odor [32]. In addition, the consumption of oxidized fish oil is associated with negative health effects, including increased oxidative stress and inflammation [33]. To ensure the nutritional quality and safety of fish oil, it is critical to understand and control the factors that influence its oxidative stability. These factors include the presence of natural antioxidants, storage conditions, and the processing methods used. Fish oils are generally stored at low temperatures, protected from light, and in tightly closed containers. Some authors proposed the use of natural antioxidants as a novel method to prevent oxidative reactions and to scavenge the peroxide radicals formed at the beginning of the oxidative reactions before the chain reactions start [34]. However, the use of natural antioxidant films based on natural biodegradable polymers that can serve as coatings or as edible film layers for fish or fish products remains scarce [35]. The current literature shows little information related to the effect of gallic acid combined with orange essential oils on the properties of the chitosan–gelatin blend aimed to be used as active packaging. Moreover, according to our survey, up to date, there has been no study on the application of active antioxidant packaging films of this type. Therefore, the aim of this work was to determine the influence of active substances, gallic acid, and orange essential oils on the physico-chemical (color, transparency, thickness, water content, solubility, and swelling capacity), barrier (permeability to oxygen, carbon dioxide, and water vapor), and antioxidant film properties (total phenols, antioxidant activity, and percentage of DPPH inhibition). The well-examined films were used as packaging lids for fish oil whose oxidation stability was measured over 30 days of storage at room temperature and expressed as k-values. 

## 2. Materials and Methods

### 2.1. Materials and Reagents

Films were made from the natural biopolymers chitosan and gelatin derived from fish (chitosan type 652, molecular weight 165 kDa, degree of deacetylation above 85%, France Chitin, Marseille, France, and gelatin from fish, Louis Francois, Croissy Beaubourg, France), gallic acid (GA, CAS 149-91-7, Sigma Aldrich, Steinheim, Germany), and orange essential oils (OEOs, Ireks aroma d.o.o., Jastrebarsko, Hrvatska) were used as a source of antioxidants. According to the producer, the principal volatiles in OEOs were sabinene (0.64%), alfa pinene (0.93%), mircene (3.44%), and limonene (91%). Vegetable glycerine (minimum purity 99.5%, E422, Dekorativna točka d.o.o., Poznanovec, Croatia) was used as a plasticizer. Distilled water and an aqueous solution of acetic acid (prepared to 2% *v*/*v*, glacial acetic acid, J.T. Baker, Schwerte, Germany) were used as solvents. Magnesium nitrate (Mg(NO_3_)_2_, Sigma—Aldrich, St. Louis, MO, USA) was used in the preparation of a saturated solution to maintain relative humidity (53% RH). 

### 2.2. Film Preparation

For film preparation, aqueous acetic acid was used to prepare chitosan dispersions (at 2% *w*/*v*), while distilled water was used for gelatin dispersions (at 4% *w*/*v*). In order to achieve the efficient gelation and polymerization of gelatin polymer chains, the solution was heated at 70 °C for 30 min on a magnetic heating plate, followed by mixing both solutions for 60 min. Then, glycerol was added at 20% *w*/*w* relative to the polymer dry weight (both chitosan and gelatin) and stirred for another 10 min. Finally, antioxidant compounds were added as follows: for the CSGELGA formulation, gallic acid was added as 5% *w*/*w* of gelatin; for the CSGELOEO 0.9% *w/v* of a filmogenic solution and for CSGELGAOEO, firstly GA (5% *w*/*w* of gelatin) and then OEOs (0.9% *w/v* of a filmogenic solution). In formulations with active agents, glycerol was added in the final step to allow GA and OEOs to bond to polymer chains. After the addition of active compounds, the filmogenic solutions were homogenized using a ULTRATURRAX (IKA T18D, Staufen, Germany) at 8000 rpm for 15 min. As a final step, filmogenic solutions were poured on a Petri dish of known dimensions and volumes, and put in a ventilated climatic chamber (HPP, Memmert, Schwabach, Germany) until dried (for 24 h at 30 °C and 40% RH). Once the solvents were evaporated, films were peeled off the surface and kept in a desiccator maintained either at 53% RH or ~0–1% RH until further analysis. All formulations are summarized in Table 1.

### 2.3. Characterization of Film Forming Solutions

The rheological properties of freshly prepared filmogenic solutions with different compositions were characterized using an RM 100 Plus Viscometer (Lamy Rheology Instruments, Champagne au Mont d’Or, France) according to the ISO 2555:2018 standard with results expressed in mPa·s. The goal was to determine the rheological parameters and solution behavior at 20 °C. The apparent viscosity (*η_a_*, Pa·s) was measured at the shear rate 5 s^−1^, and the consistency coefficient (*k*, Pa·s^n^) and flow behavior index (*n*) were calculated.

The pH was measured using a laboratory FiveGo portable pH meter (Mettler Toledo, Greifensee, Switzerland). An average of at least three measurements was taken for results. 

### 2.4. Film Characterization

#### 2.4.1. Thickness, Moisture Content, Liquid Absorption Capacity and Solubility

The thickness (μm) of each film sample was measured using a handheld micrometer (Digimet, HP, Helios Preisser, Gammertingen, Germany) to an accuracy of 0.001 mm. The mean of ten random measurements from different positions of each film was calculated.

The content of water in the film was determined by the difference between the mass after drying (*W_f_*) and the initial content of dry matter (*W_i_*). The solubility of the films was defined as the amount of dry matter dissolved in distilled water after 24 h of immersion according to the method in [36]. Before measurement, all samples were stored in a desiccator under controlled conditions of an RH of 53%. Films of equal dimensions (2 × 2 cm) were first dried at a temperature of 105 °C, then weighed on an analytical balance to determine their initial dry matter content (*Wi*) and transferred to glass vials with 30 mL of distilled water at 23 ± 1 °C. After 24 h of stirring, films were taken out of the vials and again dried at 105 °C in a dryer (Memmert, Schwabach, Germany) until constant mass, and cooled and weighed to determine the mass of dry matter not dissolved in water (*W_f_*). Film solubility (*S*, %) was calculated according to the following equation (Equation (1)):

(1) $$S\left(\%\right)=\frac{Wi-W_{f}}{W_{i}}*\;100\;\;$$

The film swelling is described by the swelling capacity (swelling ratio, *SC*), which was determined by the standard method [37]. The swelling capacity was calculated using the following equation (Equation (2)):

(2) $$SC\;\left(\%\right)=\frac{W_{s}-W_{d}}{W_{d}}*100$$

where *W_s_* is the mass of samples after swelling and *W_d_* is the mass of the dry sample.

To evaluate the liquid absorption capacity of the materials, materials were immersed in water, and 10 % and 95 % *v/v* ethanolic solutions (food simulants) were measured. Films were cut into pieces of 4 × 5 cm^2^ and weighed (*W_i_*). They were immersed in a glass containing 10 mL of water or ethanolic solution at 25 °C, then the vial was closed and stirred at 150 rpm for 48 h. The simulant remaining on the surface of the film was dried using a filter paper sheet and the film was weighed again (*W_f_*). The experiment was carried out in triplicate; the liquid absorption capacity was expressed as weight change percentage, and it was calculated by Equation (3).

(3) $$Weight\;change\;\left(\%\right)=\frac{W_{f}-W_{i}}{W_{i}}*100$$

where *W_f_* is the mass of samples after swelling and *W_i_* is the mass of the dry sample.

#### 2.4.2. Optical and Surface Properties

The color of the film was determined using a colorimeter (Konica Minolta Spectrophotometer CM3500d, Langenhagen, Germany) that works on the principle of the CIE L*a*b* spatial color diagram. An average of 10 measurements were considered. Results are expressed as *L*, *a*, *b*, and Δ*E*, where Δ*E* was defined as the total colorimetric difference referring to film without antioxidants (4).

(4) $$\Delta E=\sqrt{{\Delta L}^{2}+{\Delta a}^{2}+{\Delta b}^{2}}$$

$$\Delta L=L_{0}-L_{1}$$

$$\Delta a=a_{0}-a_{1}$$

with *L*_0_, *a*_0_, and *b*_0_ as reference values (CSGEL) and *L*_1_, *a*_1_, and *b*_1_ as values for the test film. 

The transparency of the films was measured using a UV–VIS spectrophotometer (Lambda 25, PerkinElmer, Waltham, MA, USA). The film pieces were placed at the designated place in the instrument and the absorbance was measured in the spectral range from 200 to 800 nm. The values measured at 600 nm were defined as the film transparency or *T*_600_ value, expressed as the quotient of *A*_600_ and film thickness (mm).

Fourier Transform Infrared (FTIR) spectrometry was used to study the preliminary surface structures (PerkinElmer Frontier, Beaconsfield, UK). FTIR spectra were measured in the frequency range from 4000 to 400 cm^−1^ using ATR (attenuated total reflectance) with a ZnSe crystal. For each measurement, 16 scans were performed with a resolution of 4 cm^−1^. The spectra were measured in duplicate. The aims of this analysis were to determine the changes induced by mixing chitosan and gelatin and the incorporation of gallic acid into the mixed polymer chains at the molecular level.

#### 2.4.3. Barrier Properties (Permeability to Oxygen, Carbon Dioxide and Water Vapour)

The permeability of the films to O_2_ and CO_2_ was measured using the manometric method (GDP-C, Brugger Feinmechanik GmbH, München, Germany). The results are presented as oxygen and carbon dioxide permeability coefficients (*P*O_2_ and *P*CO_2_) expressed in cm^3^ m^−1^ day^−1^ Pa^−1^. The water vapor permeability of the films was determined gravimetrically using a modified [38] standard method adapted for edible materials and at an RH differential of 75% [39]. Prior to measurement, samples were stored in a desiccator under controlled humidity conditions (53% RH). The results are reported as water vapor permeability (*WVP*) and water vapor transmission rate (*WVTR*).

#### 2.4.4. Determination of Total Phenolic Content and Antioxidant Activity 

For the preparation of samples, 0.2 g of dry films were stirred overnight in distilled water (20 mL) to extract active compounds. Solutions were filtered and the remaining supernatant was taken for analysis. 

The proportion of total polyphenols in the films was determined by a method based on a colorimetric reaction with the Folin–Ciocalteu reagent [40]. In this method, phenolic compounds are characterized by the fact that when they react with the Folin–Ciocalteu reagent, a mixture of phosphotungstic and phosphomolybdic acid, a blue-colored complex, is formed which is proportional to the concentration of phenol in the solution. The sample (100 μL), Folin–Ciocalteu reagent (200 μL), and 2 mL of deionized water were pipetted into a glass test tube. After 3 min, a saturated sodium carbonate solution (1 mL) was added, vortexed, and thermostated for 25 min at 50 °C. After that, the absorbance was measured at a wavelength of 765 nm.

The DPPH (1,1-diphenyl-2-picrylhydrazyl) method is based on the capture of free radicals [40]. The exact mass of film samples (around 0.2 g) was placed in glass tubes and immersed in a 0.004% DPPH solution for 30 min at room temperature in the dark. When DPPH radicals react with antioxidants, they become stable molecules, which causes a change in color from dark purple to pale yellow. A sample’s antioxidant activity is inversely proportional to the intensity of the purple coloration of DPPH. The initial and control test was the DPPH solution itself (*A_controlDPPH_*). After 30 min, the absorbance at 517 nm was measured. The percentage of inhibition was calculated according to Equation (5):

(5) $$\%\;\mathrm{inhibition}=\frac{A_{control\;DPPH}-A_{sample}}{control}\times 100\%$$

The FRAP (Ferric Reducing Antioxidant Power) method was used to determine the amount of total polyphenols [40]. This method is based on the ability of antioxidant compounds to reduce iron (III)-tripyridyl-triazine to iron (II)-tripyridyl triazine. A decrease in color intensity occurs because of the reaction, which is proportional to the antioxidant concentration. The FRAP reagent was prepared by mixing 25 mL of acetate buffer (0.3 M), 2.5 mL of a TPTZ (2,4,6-tripyridyl-*s*-triazine) reagent, and 2.5 mL of iron (III) chloride in a ratio of 10:1:1. The total of 300 μL of extracted film solution and 2250 μL of FRAP reagent was pipetted into glass tubes, mixed, and thermostated for 10 min at a temperature of 30 °C in a water bath. After the reaction, the absorbance was measured at a wavelength of 593 nm. The blank contained only the FRAP reagent and distilled water.

### 2.5. Evaluation of Fish Oil

**Figure 1:**
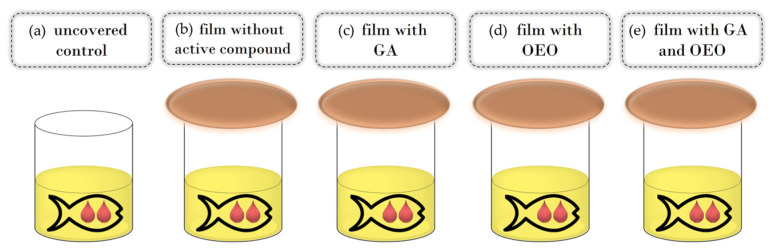
Schema of the experimental setup: control (**a**), CSGEL film without active compounds (**b**) and CSGEL films enriched with GA (**c**), OEOs (**d**), and both (**e**). CSGEL—chitosan/gelatin; GA—gallic acid; OEO—orange essential oil.

Commercial cod liver fish oil (25 mL) was put in glass cups that were covered with active films in such a way that the film was in contact with the external atmosphere on one side and in contact with the internal headspace of the glass cell (on the lower side). Tightness was assured with screw cup covers. For control samples, fish oil was uncovered or in other words, directly exposed to ambient air (Figure 1). All experiments were run in triplicate. Samples were kept in the dark at an ambient temperature (20 ± 2 °C) for 30 days. The sampling was performed at the beginning, after 7 days, after 14 days, and after 30 days. For each sampling time, new samples were used; thus, there was no influence of the changes in the inner headspace composition due to the opening of the cups. 

The oxidative status of fish oil samples was assessed by the spectrophotometric determination of conjugated dienes and trienes according to the official method of the International Olive Council [41]. Briefly, an aliquot of 0.25 g of fish oil was weighed in a 25 mL volumetric flask and dissolved in isooctane to obtain a solution concentration of 1 g of oil/100 mL of isooctane. As the method requires extinction coefficients in the range of 0.1–0.8, the obtained solution was further diluted by isooctane to a concentration of 0.1 g/100 mL. Extinction was measured in 10 mm optical path quartz cell at 232 and 268 nm and specific extinction coefficients (K232 and K268) were calculated as follows (Equation (6)):

(6) $$K\lambda =\frac{E\lambda }{c\;*\;s}$$

where:

*Kλ* = specific extinction at wavelength *λ*;
*Eλ* = extinction measured at wavelength *λ*;
*c* = concentration of the solution in g/100 mL;
*s* = path length of the quartz cell in cm

### 2.6. Statistical Analysis

Statistical analyses of the data were performed using Xlstat-Pro (win) 7.5.3. (Addinsoft, New York, NY, USA). One-way analysis of variance (ANOVA) and Tukey’s multiple comparison tests were performed for all data, and statistical differences were evaluated on the ranks. Significant results are determined at a confidence level of *p* > 0.05.

## 3. Results

### 3.1. Characterization of Film Forming Solutions (FFS)

The pH values of the various mixed solutions are given in Table 2. As expected, CS (4.63 ± 0.01, result not given in the table) had lower pH than GEL (5.44 ± 0.01, result not given in the table), while the mixture was somewhere in between (from 4.56 to 4.75). According to some authors, stable chitosan–alkaline gelatin complexes can exist in pH ranges between 4.7 and 6.7 which correspond to the pI and p*K*_a_ of alkaline gelatin and chitosan, respectively. CSGEL solutions without antioxidants had pH values within this range, however, the addition of GA and OEOs led to a further drop in the pH values. According to others, chitosan–gelatin polyelectrolyte complexes can also be formed at an acidic pH because highly charged chitosan causes the additional ionization of gelatin carboxyl groups [42,43]. 

**Figure 2:**
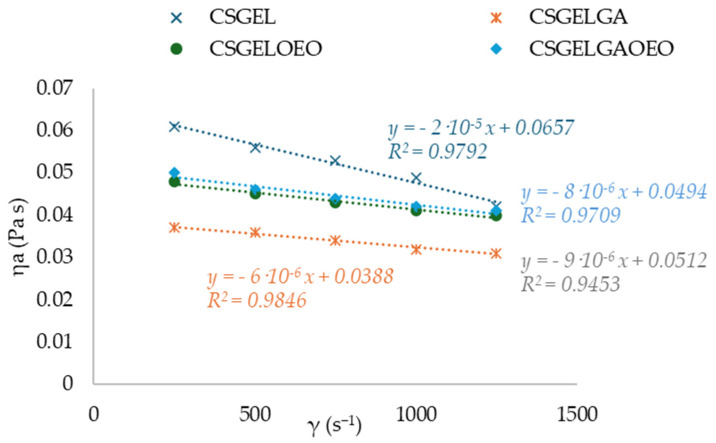
Curves of apparent viscosity *η_a_* (Pa s) vs. shear rate *γ* (s^−1^) for various CSGEL solutions. CSGEL—chitosan/gelatin; GA—gallic acid; OEO—orange essential oil.

Knowledge of the flow behavior of film-forming solutions is important to create a dry film that is thick, uniform, and spreadable. The results of viscosity for all tested film-forming solutions showed a thinning behavior over a wide range of shear tests, often describing polymer melt behavior (Figure 2). The *n* and *K* parameters were calculated with the classical power law model. The *n* index ranged between 0.77 and 0.89, indicating the non-Newtonian nature of the sample, resulting from the entanglement between molecules. These results were within the same range as previously given for CSGEL (mixed in ratio of 2:1) [43]. A similar phenomenon has been previously reported for various CS and its derivative solutions [44]. In chitosan–gelatin mixtures, the gelatin macromolecules interact with the positively charged amino groups in chitosan and block them. In chitosan salt solutions, anti-ions of low molecular weight [43] screen positively charged amino groups and chitosan salt solutions [45,46]. It seems that the screening of chitosan-positive charges by gelatin macromolecules favors the rapprochement of their links at distances suitable for intramolecular bonding. The k value was the highest in the CSGEL solution, while with the addition of both GA and OEOs the k values decreased. This decrease suggests that the gel network became weaker due to the formation of new interactions between the polymers and the phenolic groups from gallic acid and OEOs. This behavior is similar to that previously reported for CSGEL and pomegranate peel extract [47]. The GA may form phenolic dimers or trimers or intermolecular hydrogen bonds, damaging the original tight structure of the CSGEL matrix or it may form a covalent bond with CS or GEL polymers that are not bound together. As a result, the intramolecular and intermolecular interactions of FFS were reduced, which eventually led to a lower viscosity of the solution [47,48,49,50,51]. An increase in viscosity with the addition of OEOs compared to CSGELGA was possibly due to the reduced interaction of GA with CS and GEL, as GA and OEOs compete for binding to free polymer chains, thus increasing the viscosity. 

### 3.2. Film Characterization

#### 3.2.1. Thickness, Moisture Content, Liquid Absorption Capacity, and Solubility 

Table 3 shows the moisture content, liquid absorption capacity, and solubility in the developed films. Film thickness (Table 3) did not change significantly depending on the film formulation. The control CSGEL film had the highest solubility, while the addition of gallic acid and OEOs decreased the moisture content, probably due to their hydrophobic character. In addition, for formulations with GA, it is possible that GA acting as a crosslinker gives a film with a much denser structure and less free space for binding water molecules. Extensive cross-linking between the constituents prevents their interaction with water and hence contributes to a lesser degradation percentage in the case of films with gallic acid with respect to their counterparts. A similar was found for chitosan–starch–gallic acid ternary films [52]. Liquid absorption capacity in various food stimulants is also given in Table 3. In the swelling polymer systems, water diffusion and polymer chain relaxation have been defined by the relative rates of diffusion and polymer relaxation [53]. Surprisingly, water uptake was higher in the samples with gallic acid and OEOs. It is possible to explain higher swelling values by the reduction in crystallinity observed with the addition of orange essential oils or by a higher water content of these samples. According to [54], a decrease in crystallinity leads to the greater availability of hydrophilic groups and consequently higher swelling. All samples showed a significant increase in weight after the immersion in water (from 178 to 440%) and 10% EtOH (up to 2000%), while sample weight loss was observed after the immersion in 96% EtOH. The weight loss of active materials in fatty food stimulants was attributed to the high ethanol solubility of active complexes and dehydration of gelatine in ethanol [55]. In addition, dehydration by ethanol precipitation might lead to a more orderly internal structure and more stable films afterward [56].

#### 3.2.2. Barrier Properties

Even though no significant difference in *WVTR* values among samples was measured, the *WVP* of films with GA or OEOs was lower than that of the control CSGEL film. This was probably due to the cross-linking effect of GA and increased hydrophobicity (lower moisture content) after the addition of GA or OEOs (Table 4). The decrease occurred due to changes in the structure of the polymer, hindering the diffusion of water molecules, as well as hydrophobic reactions and hydrogen bonds that can be formed between polyphenols and polar groups of biopolymers. The *WVTR* was in the magnitude of the order of 10^−2^ g m^−2^ s^−1^, while the *WVP* values ranged in the magnitude of the order of 10^−10^ g m^−1^ s^−1^ Pa^−1^. Values were higher than those given in the literature [57,58], and these differences were attributed to different measuring conditions, since the mentioned authors performed the analysis at the ΔRH of 50%, while in the present study, it was performed at the ΔRH of 75%. At a higher RH differential, the hydrophilic nature of gelatin allowed higher quantities of water vapor to migrate through the film. Moreover, water vapor transport through the material is influenced by the ratio of hydrophilic/hydrophobic film components, film crystallinity, path tortuosity, and the presence of surface or structural defects [59,60,61]. As a result of combining both additives (CSGELOEOGA film), water vapor permeability increased. This may be due to the less crystalline polymer structure in the CSGEL matrix after the addition of both compounds, which resulted in a lower uniformity of the film [62]. Low *WVP* values prevent moisture from escaping into the environment, which can cause drying and degrade the quality and structure of the product. Results of gas permeability measurements are given in Table 4.

Packs containing oxygen negatively impact the quality and shelf-life of oxygen-sensitive food products, as it leads to various oxidation reactions that can result in off-flavor production, loss of nutritional value, and color change. Generally, gelatin films are recognized as having less gas barrier properties than chitosan films. It is therefore clear that the presence of chitosan can explain the gas barrier performance of mixed film formulations such as those used in this study. There were no significant differences among samples in gas barrier properties in measured conditions, with all values in the order of 10^−5^ cm^3^ m^−1^ d^−1^ bar^−1^. It is somewhat difficult to compare results with those of the scientific literature, since many factors, such as polymer packing, concentration, additive type, concentration, and measuring conditions, have a significant influence on the results. For example, in a previous study [63] it was reported that CSGEL blends had permeability coefficients also in the range of 10^−5^ cm^3^ m^−1^ d^−1^ bar^−1^, even though films were prepared with a different CS/GEL ratio; however, with the addition of gallic acid significant changes occurred which was not observed in the present study. There may have been more significant changes and disruptions in the crystalline structure of the film matrix as a result of a higher GA to CS and GEL ratio.

#### 3.2.3. Optical and Spectrophotometric Properties

**Figure 3:**
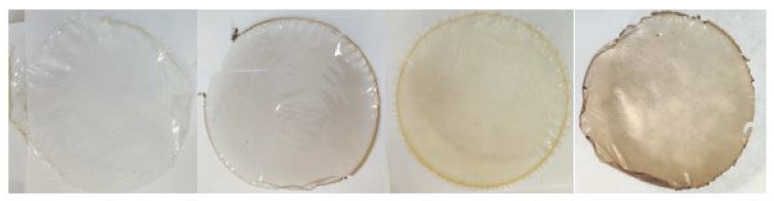
Picture of produced films (from left to right, CSGEL, CSGELGA, CSGELOEO, CSGELGAOEO). CSGEL—chitosan/gelatin; GA—gallic acid; OEO—orange essential oil.

All films had a coherent visual appearance without cracks or failures. For the CSGEL, the film color varied from clear to transparent, while when OEOs were added, the film color changed from transparent to light yellow to light green. Thus, the CSGEL served as a referent film for the calculation of the total color difference (Δ*E*) (Table 5). By adding gallic acid, the film had developed a violet taint and was described as darkened (Table 5, Figure 3).

Accordingly, colorimetric results (Table 5) indicate that the CSGEL had significantly the highest L* value, which was decreased after the addition of both functional compounds, being the lowest for samples with gallic acid. The b* parameter was negative for the CSGEL sample. For other films, higher and positive values indicated increased yellowness, especially for formulations with OEOs. Positive a* values indicated red/violet tones in formulations with GA. Moreover, the most significant color difference was for the formulation, with both compounds reaching > 20 compared with the native control film.

Since consumers need naturalness, it is imperative to measure the opacity of films that cover products without altering their appearance [64,65]. As low a value as possible is ideal when measuring transparency; the lower the value, the more transparent the sample [66]. OEOs added to film formulations made those films opaquer, which significantly influenced their transparency [67].

**Figure 4:**
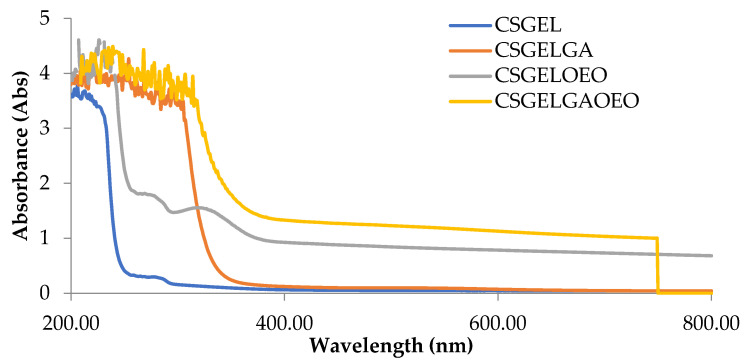
Absorbance of films in the range from 200 to 800 nm. CSGEL—chitosan/gelatin; GA—gallic acid; OEO—orange essential oil.

The absorption spectra of all film formulations, in the UV and visible range of the spectrum, are given in Figure 4. It is generally recognized that gelatin-based films are effective ultraviolet barriers because they contain high amounts of amino acids with aromatic groups in the side chains that absorb UV radiation at wavelengths of up to 280 nm [66]. The addition of gallic acid reduced the transmittance in the whole UV spectra, with significant differences for CSGELGA up to 300 nm (for 400%) and even up to 320 nm for CSGELGA OEO (for 500%). With the addition of the orange essential oils (CSGELOEO), the absorbance was significantly reduced up to 370 nm when compared to the control CSGEL film. The observed results follow trends given in the scientific literature for films cross-linked with anillin [67], betel (*Piper betle* L.) leaf ethanolic extracts [58], and green tea carbon dot [68], indicating a good UV protection of produced formulations, which could very likely be used in the future to reduce oxidation changes and extend the shelf-life of the product.

**Figure 5:**
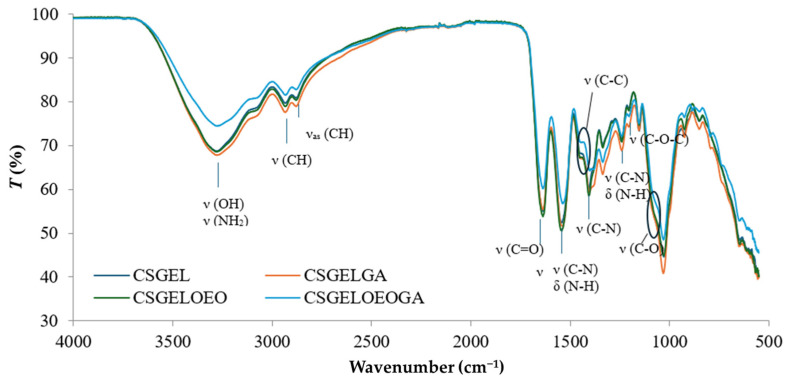
FTIR spectra of various chitosan and gelatin film formulations. CSGEL—chitosan/gelatin; GA—gallic acid; OEO—orange essential oil.

FTIR spectra of all films are given in Figure 5.

The characteristic IR bands of chitosan and gelatin were observed in all formulations. The broad peak between 3500 and 3000 cm^−1^ was attributed to stretching vibrations of the -OH groups while the stretching vibrations of -NH groups were overlapped. The peaks observed at 2933 and 2881 cm^−1^ were attributed to CH stretching vibrations. Moreover, the wavelengths of the bands differ from the previously described characteristic polymer peaks (CS and GEL with peaks at 2923 and 2878 cm^−1^ for CS and 2937 and 2879 cm^−1^ for GEL, respectively) [63]. These shifts suggested that the chitosan and gelatin molecules interacted by forming hydrogen bonds between N-H and O-H groups. The carbonyl groups of fish gelatin interact ionically with the oppositely charged amine groups (NH_3_^+^) of chitosan in acidic conditions. Furthermore, many functional groups, such as -COOH, -NH_2,_ and -OH groups, in gelatin interact with the -OH and -NH_2_ groups present in chitosan chains [69]. Thus, the interactions between fish gelatin and chitosan are attributed to secondary interactions (electrostatic interactions and hydrogen bonds). The characteristic peaks at about 1637 cm^−1^, 1536 cm^−1^, and 1240 cm^−1^ were ascribed to amide I, amide II, and amide III, respectively [70,71]. No differences in peak position between samples were observed in the amide I and amide III regions, however, the intensity was lower in samples without gallic acid, possibly an indication of crosslinking that generally occurs as amide and ester linkages (at 1740 cm^−1^) [72]. The latter was not visible in the present study. However, there was a shift in amide II from 1541 cm^−1^ in CSGEL and CSGELOEO to 1535 cm^−1^ in samples with GA. These results suggest an interaction with gallic acid, and possibly crosslinking. Amino and hydroxyl groups involved in complexation could initiate stronger intermolecular interactions through hydrogen bonding. The C–N stretching vibration of chitosan at around 1411 cm^−1^ [63] was shifted to 1405 cm^−1^, also indicating surface structural changes. Also, the typical stretching vibrations of the C–C bonds of the aromatic ring of gallic acid, which usually appear at 1441 cm^−1^, were shifted, and appeared only in GA-enriched samples as a knee around 1386–1429 cm^−1^, indicating the bonding of GA.

The band at 1153 cm^−1^ was attributed to the asymmetric stretching of the C–O–C bridge in chitosan. The characteristic signal of the C–O stretching vibration of chitosan at 1066 cm^−1^ was broadened and appeared as shouldering around 1050 cm^−1^, while that at 1028 cm^−1^ was present in CSGEL and was shifted to 1030 cm^−1^ and 1031 cm^−1^ with the addition of antioxidants. This band is a characteristic vibrational band of the benzene ring. The signal at 850 cm^−1^ corresponds to the CH bending out of the plane of the ring of monosaccharides [73]. In the samples with gallic acid, bands were also observed at 795 cm^−1^ and 743 cm^−1^.

#### 3.2.4. Antioxidant Activity

Antioxidant activity plays an important role in determining the effectiveness of active films because it indicates how well they can prevent or delay the oxidation of treated products [74,75,76]. The results for the total phenolic content and antioxidant activity are given in Table 6.

**Figure 6:**
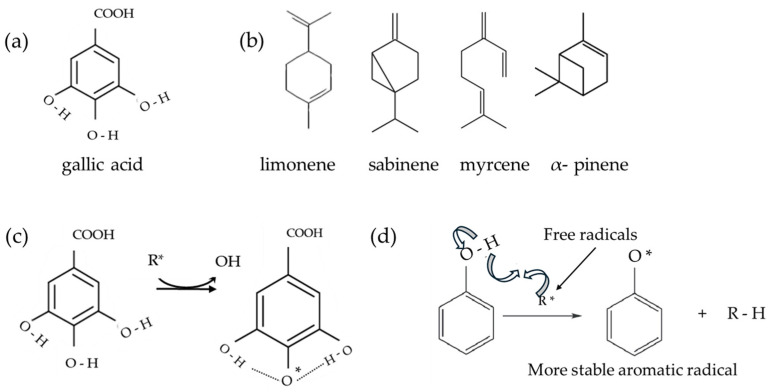
Schema of (**a**) gallic acid; (**b**) principal orange essential oil compounds; (**c**) possible stabilization of gallic acid; and (**d**) schema of a possible stabilization of aromatic ring—oxidation of antioxidants. Schemas adapted from [78,79,80,81].

Even though neither chitosan nor gelatin contains phenols, the results show some phenolic content that is related to the reaction of the FC reagent with the amino group of chitosan [77]. The total phenolic content was the highest in the CSGELGA film. It seems that the measured quantity of GA was about 50% lower than initially added to FFS before film drying. This was partially attributed to the bonded GA in the CSGEL structure, shown also by FTIR analysis and as evidence of the cross-linking effect of GA. This might indicate that during sampling for AO analysis, the added GA was not completely released in the solvent media and must be considered as such. The scientific literature [78,79] gives different pathways of antioxidant activity of gallic acid and sabinene, myrcene, pinene, and limonene as principal compounds of orange essential oils, thus a schema giving some possible pathways is given in Figure 6. According to FRAP analysis, CSGELGA has the highest antioxidant capacity, followed by films with GA/OEOs and OEOs. However, the antioxidant capacity calculated as the % of DPPH inhibition showed that the CSGELGAOEO has the highest AO potential. Differences in results from both methods are related to the different mechanisms of action of the assays; while FRAP evaluates the ferric-reducing power, the DPPH method measures the ability of antioxidants to donate an electron. Moreover, the methodology also differs since the DPPH scavenging was tested on the films as a whole and FRAP on the film extracts.

### 3.3. Fish Oil in Protective Atmosphere with Antioxidant Film

**Figure 7:**
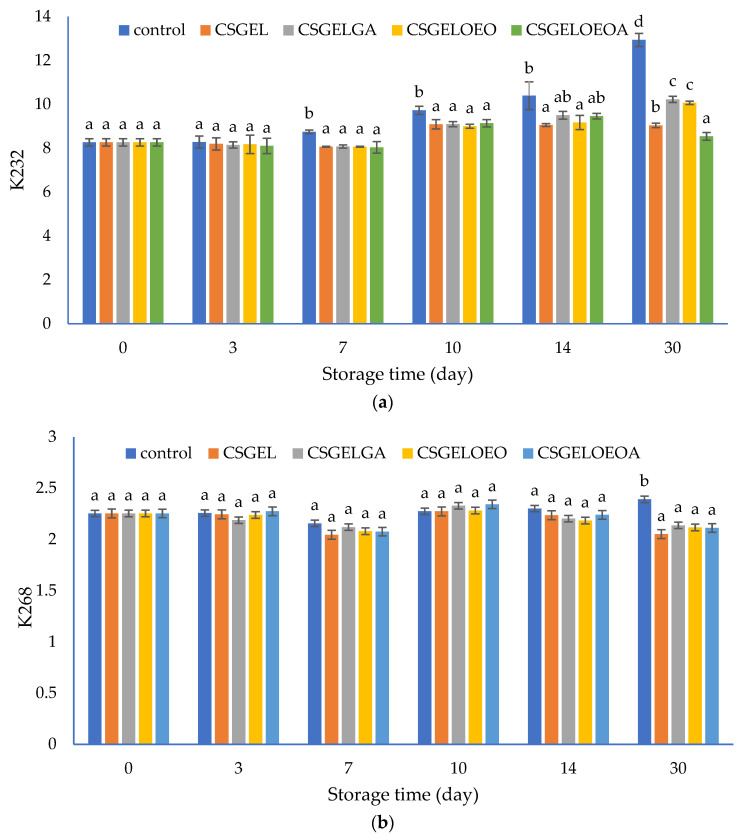
The evolution of (**a**) conjugated dienes (K232) and (**b**) conjugated trienes (K268) values in different oil samples covered with various films during 30 days of storage at 20 ± 2 °C. CSGEL—chitosan/gelatin; GA—gallic acid; OEO—orange essential oil. Different exponents (^a–d^) indicate significant differences among samples on each sampling day (*p* < 0.05).

The content of conjugated dienes (K232) and conjugated trienes (K268) were measured spectrophotometrically. These compounds are produced due to the rearrangement of the methylene-interrupted double bonds during the oxidation of PUFA and are used as an indicator of the oxidative status of lipids [80]. The results are given in Figure 7. The increased UV absorption of K232 and K268, in conjunction with conjugated diene and triene formation, is proportional to oxygen uptake and results from peroxide formation. Values of these parameters can be used as relative measurements of lipid oxidation that, when present even at very low levels, significantly impair the taste and odor of the oils [81].

In this study, the K232 value of control samples progressively increased from the initial value of 8.26 to 12.94 at the end of the 30-day storage period. Analysis was performed at ambient temperature to evaluate the oil behavior as it should happen in real-life scenarios and households. Samples protected by films showed significantly lower K232 values compared to the control sample from the seventh day until the end of the storage period, with the exception of CSGELGA and CSGELOEOA which showed no significant differences to all other samples on day 14. At the end of storage, the lowest values were observed for samples packed in CSGELOEOA films (K232 = 8.54), followed by CSGEL (K232 = 9.04), and CSGELOEO and CSGELGA films (K232 = 10.07 and K232 = 10.22, respectively). The K268 values showed very small changes throughout the whole experiment, indicating slight but statistically significant differences between control and packed samples only at the end of the storage experiment (day 30). Such differences are expected since in the oxidation of omega-3, PUFA conjugated dienes are readily formed, and conjugated trienes form in lower amounts due to the further oxidation of conjugated diene products, which was confirmed in several previous studies [82].

The lower oxidation of oil in samples with films was attributed to the absence of oxygen throughout the storage period, due to good oxygen barrier properties (Table 3) that did not allow the passage of oxygen in the headspace from the external atmosphere. In addition, in active formulation, the presence of antioxidants led to the scavenging of oxygen in the product headspace, which can explain the effectiveness of active films against fish oil oxidation during 30 days of storage at ambient temperature (Figure 7). The most significant impact was found in formulation with combined antioxidants indicating their synergistic effect. Similar findings were previously given by [35], who found that carboxymethyl cellulose films enriched with cold-pressed green coffee oil significantly reduced peroxide value and thiobarbituric acid reactive substances in fish oil samples covered by active films and packed in an inert headspace. The effect was even more significant than in the present study, however, the authors used accelerated testing at elevated temperatures (40 °C for 16 days) and an inert atmosphere, while in the present study, the atmospheric scenario was considered. In addition, the authors mentioned that the overall antioxidant activity in similar films resulted from the hydrogen atom donation mechanism as well as due to the generation of other prooxidant phenols via electron donation [83]. Contrarily to the K232 values, significant changes in K268 were observed only after 30 days of storage, when significantly higher values were observed for the control sample, while no significant differences between the samples protected by films were observed (Figure 7b).

## 4. Conclusions

In the present study, polyelectrolyte complexes from chitosan and gelatin were produced as standalone biobased films and they were enriched with natural antioxidants, gallic acid and orange essential oils, added as single compounds or in combination. As anticipated, the addition of antioxidants substantially enhanced the functionality of the films, showing synergistic effects and yielding DPPH inhibition rates as high as 88%. Furthermore, the incorporation of gallic acid-induced cross-linking effects, as evidenced by reduced moisture content, solubility, and liquid absorption in the films. Additionally, shifts in the FTIR spectral bands suggested interactions with polymer chains, resulting in noticeable improvements in film coloration and enhanced UV barrier properties, an essential feature for preserving UV-degradable food components. The efficacy of these formulations was evaluated by applying them to fish oil as an active antioxidant and oxygen scavenging film, indirectly, over a 30-day period at ambient temperature. An increase in oxidation parameters (K232 value) was observed after 7 days of storage for the control sample, and values progressively increased by the end of storage at 30 days. However, further research on higher temperatures might lead to more significant changes and is planned. The lower oxidation rates of oil in samples with films were attributed to their excellent oxygen barrier properties, which prevented the ingress of oxygen from the external atmosphere and exhibited scavenging activity. This study offers a comprehensive characterization of biobased film formulations with antioxidant properties presenting a viable solution for preserving the quality and extending the shelf life of fish oil, an invaluable component of fresh fish. Further studies focusing on fish oil-rich samples, such as fresh fish, are envisaged to further explore the potential applications of these formulations.

## Figures and Tables

**Table 1:** Sample composition and sample coding (abbreviation).

Abbreviation	Chitosan (CS) (%, *w*/*v*)	Gelatin (GEL) (%, *w*/*v*)	Glycerol (%, *w*/*v*)	Gallic Acid (GA) (%, *w*/*v*)	Orange Essential Oil (OEO) (%, *w*/*v*)
CSGEL	2	4	1.2	0	0
CSGELGA	2	4	1.2	0.2	0
CSGELOEO	2	4	1.2	0	0.9
CSGELGAOEO	2	4	1.2	0.2	0.9

**Table 2:** pH and viscosity parameters of filmogenic solutions.

Sample	pH	Viscosity Parameters Power Law Model		*µ*_p_ (Pa s)
		*n*—Flow Index	*k* (Pa s ^n^)	
CSGEL	4.75 ± 0.01 ^a^	0.77 ± 0.04 ^b^	0.2195 ± 0.0109 ^a^	0.042 ± 0.002 ^a^
CSGELGA	4.63 ± 0.01 ^b^	0.89 ± 0.08 ^b^	0.0679 ± 0.0882 ^c^	0.031 ± 0.007 ^b^
CSGELOEO	4.56 ± 0.01 ^d^	0.89 ± 0.11 ^b^	0.0897 ± 0.0802 ^b^	0.040 ± 0.005 ^a^
CSGELGAOEO	4.59 ± 0.00 ^c^	0.88 ± 0.04 ^b^	0.0988 ± 0.0034 ^b^	0.041 ± 0.002 ^a^

Different exponents (^a–d^) within the same column indicate significant differences among samples (*p* < 0.05). CSGEL—chitosan/gelatin; GA—gallic acid; OEO—orange essential oil.

**Table 3:** Moisture content (*M*), solubility (*S*), and liquid absorption capacity (*SC*).

Sample	*M* (*%*)	*S* (*%*)	*SC* (*%*)		
			H_2_O	96% EtOH	10% EtOH
CSGEL	15.06 ± 0.93 ^a^	45.96 ± 0.18 ^a^	178.28 ± 0.56 ^a^	−17.57 ± 0.93 ^b^	1976.92 ± 96.14 ^d^
CSGELGA	11.46 ± 4.14 ^a^	32.37 ± 0.06 ^c^	264.34 ± 18.05 ^b^	−11.94 ± 0.19 ^a^	727.25 ± 20.99 ^b^
CSGELOEO	12.25 ± 1.71 ^a^	36.69 ± 0.13 ^b^	441.09 ± 5.72 ^c^	−25.81 ± 5.58 ^c^	1487.74 ± 72.14 ^c^
CSGELGAOEO	12.68 ± 0.34 ^a^	32.59 ± 0.14 ^c^	204.28 ± 14.90 ^b^	15.33 ± 2.63 ^b^	627.17 ± 40.05 ^a^

*WVP*—water vapor permeability; *WVTR*—water vapor transmission rate; EtOH—ethanol solution. CSGEL—chitosan/gelatin; GA—gallic acid; OEO—orange essential oil. Different exponents (^a–d^) within the same column indicate significant differences among samples (*p* < 0.05).

**Table 4:** Thickness (*l*), water vapor barrier parameters (*WVP* and *WVTR*), and gas permeability coefficients (*P*O_2_ and *P*CO_2_) of prepared films.

Sample	*l* (µm)	*WVP* $(g\;m^{-1}$ $s^{-1}$ ${Pa}^{-1}$ $)\;\times \;10^{-10}$	*WVTR* $\;(g\;m^{-2}$ $s^{-1}$ $)\;\times \;10^{-2}$	Gas Permeability Coefficients × 10^−5^ cm^3^ m^−1^ d^−1^ bar^−1^	
				*P*O_2_	*P*CO_2_
CSGEL	73.67 ± 15.81 ^a^	3.76 ± 0.09 ^a^	1.10 ± 0.05 ^a^	6.41 ± 4.10 ^a^	5.28 ± 0.75 ^a^
CSGELGA	62.57 ± 16.59 ^a^	2.79 ± 0.18 ^b^	1.12 ± 0.27 ^a^	6.04 ± 0.83 ^a^	5.09 ± 2.57 ^a^
CSGELOEO	60.29 ± 20.3 ^a^	2.85 ± 0.13 ^b^	1.06 ± 0.06 ^a^	6.69 ± 2.23 ^a^	6.20 ± 1.79 ^a^
CSGELGAOEO	68.57 ± 13.3 ^a^	5.98 ± 0.63 ^c^	0.99 ± 0.01 ^a^	8.30 ± 1.77 ^a^	4.26 ± 0.51 ^a^

CSGEL—chitosan/gelatin; GA—gallic acid; OEO—orange essential oil. Different exponents (^a–c^) within the same column indicate significant differences among samples (*p* < 0.05).

**Table 5:** CIELab color parameters, total color difference, and opacity (*O*) measured at 600 nm.

Sample	L*	a*	b*	Δ*E*	*O*_600_
CSGEL	90.29 ± 0.48 ^a^	0.84 ± 0.37 ^c^	−2.55 ± 1.17 ^c^	0.00 ± 0.00 ^c^	0.36 ± 0.04 ^a^
CSGELGA	79.69 ± 3.88 ^c^	6.78 ± 1.81 ^a^	−0.36 ± 1.37 ^b^	12.44 ± 4.49 ^b^	0.94 ± 0.08 ^b^
CSGELOEO	88.97 ± 0.69 ^b^	−0.25 ± 0.42 ^d^	1.99 ± 1.92 ^b^	4.83 ± 2.07 ^b^	18.49 ± 1.85 ^c^
CSGELGAOEO	71.39 ± 6.01 ^c^	8.48 ± 2.08 ^b^	5.69 ± 2.73 ^b^	21.98 ± 6.91 ^a^	17.75 ± 1.80 ^c^

CSGEL—chitosan/gelatin; GA—gallic acid; OEO—orange essential oil. Different exponents (^a–d^) within the same column indicate significant differences among samples (*p* < 0.05).

**Table 6:** Total phenolic content (*TPC*) and antioxidant capacity.

Sample	TPC (mg GAE/g Film)		Antioxidant Capacity	
	Distilled H_2_O	EtOH	FRAP (mgAAE/g Filma)	*%* Inhibition DPPH
CSGEL	1.30 ± 0.09 ^a^	nd	0.21 ± 0.05 ^d^	5.85 ± 0.09 ^d^
CSGELGA	33.35 ± 0.76 ^d^	16.44 ± 1.16 ^b^	12.82 ± 0.96 ^a^	77.86 ± 0.06 ^c^
CSGELOEO	3.30 ± 0.24 ^b^	9.71 ± 1.66 ^a^	1.79 ± 0.19 ^c^	25.69 ± 1.79 ^b^
CSGELOEOGA	20.40 ± 1.69 ^c^	27.16 ± 6.11 ^c^	8.36 ± 1.23 ^b^	88.08 ± 0.25 ^a^

Different exponents (^a–d^) within the same column indicate significant differences among samples (*p* < 0.05), nd—not detectable. CSGEL—chitosan/gelatin; GA—gallic acid; OEO—orange essential oil.

## Data Availability

All of the data is contained within the article.
